# Psychiatric Symptoms due to Thyroid Disease in a Female Adolescent

**DOI:** 10.1155/2014/972348

**Published:** 2014-11-04

**Authors:** Nelly Capetillo-Ventura, Inmaculada Baeza

**Affiliations:** ^1^Department of Psychiatry, “Dr. José Eleuterio González” University Hospital, UANL, Avenida Francisco I. Madero and Avenida Gonzalitos, Mitras Centro, 64460 Monterrey, NL, Mexico; ^2^Department of Child and Adolescent Psychiatry and Psychology, Institut Clínic de Neurociencies, Hospital Clínic, IDIBAPS, CIBERSAM, Barcelona, Spain

## Abstract

The hypothalamic-pituitary-thyroid axis is involved in the production of thyroid hormone which is needed to maintain the normal functioning of various organs and systems, including the central nervous system. This study reports a case of hypothyroidism in a fifteen-year-old female adolescent who was attended for psychiatric symptoms. This case reveals the importance of evaluating thyroid function in children and adolescents with neuropsychiatric symptoms.

## 1. Background

The hypothalamic-pituitary-thyroid axis is involved in the production of thyroid hormone which is needed to maintain the normal functioning of various organs and systems, including the central nervous system [1]. Both hypothyroidism and hyperthyroidism can cause symptoms attributable to psychiatric illness. The lack of pathognomonic symptoms to differentiate thyroid disease from psychiatric disorders makes it difficult to distinguish between the two conditions in some cases [2].

Hypothyroidism is the most common abnormality of thyroid function in children and adolescents [3]. The prevalence of thyroid dysfunction between 11 and 18 years is 1% and the most common cause of acquired hypothyroidism is chronic lymphocytic thyroiditis (Hashimoto's thyroiditis), with a 2 : 1 female predominance, and, secondly, endemic goiter due to iodine deficiency. Hypothyroidism in children can be classified as primary or secondary (central) and can be congenital or acquired and transient or permanent [4].

Most adults with Hashimoto's thyroiditis are asymptomatic [5]. There are few published articles about this issue in children and adolescents. This paper reports a case of hypothyroidism in a female adolescent who was attended for psychiatric symptoms.

## 2. Case Report

A fifteen-year-old female adolescent was admitted to the child and adolescent acute psychiatric unit of our hospital for the first time after remaining isolated in her home for four months. She had initiated treatment in the outpatient clinic of her district in June 2013, attending only one visit and refusing all psychiatric treatment thereafter.

There was a family history of schizophrenia in second degree relative on her mother's side. The patient's medical history, according to her family, included recurrent acute bronchitis until 10 years of age. No history of alcohol or tobacco consumption or psychoactive substance use was reported. The patient was very selective and restrictive in her meals since her childhood. She only ate biscuits, ham croquettes, bread, and omelettes.

Regarding school, she described acceptable performance until the previous academic year when she presented some difficulties, and her teachers decided to change her to another classroom with a lower academic level.

Her premorbid personality was described as quiet and shy, with some difficulty in social relationships, but with a group of friends at the beginning of the episode. She lived with her parents and an 18-year-old sister in a mid-size city (with a population of about 216,000 inhabitants).

The patient reported that during the previous academic year she had been separated from her friends due to the change of classroom and at the same time she had begun to suffer digestive cramps and headaches, and as a result she stopped going to school. She stayed at home, with no interest in leaving the house, playing computer games and surfing the internet all day from April 2013 until her admission in our department 6 months later. The family explained that she had also become withdrawn from them, showing little interest in conversation, giving only monosyllabic answers, and gesturing in response to her parents' questions. She showed neglect in her personal hygiene and apathy regarding her physical appearance.

At the time of her admission, the mental status examination showed she was conscious and oriented in time, place, and person, though unkempt and exhibiting psychomotor retardation, poor eye contact, and limited language, answering questions with monosyllables or gestures. She also displayed emotional lability when discussing sensitive issues like friends and school and showed hypothymia, poorly reactive mood, apathy, weakness, and anhedonia and was only interested in computer usage. She did not exhibit impaired sensory perception, self-referentiality, or psychotic behavior, though she was sleeping from 4:00 am to noon. Her food intake was very selective, limited to certain specific foods, but there was no loss of appetite. The patient denied having suicidal ideation.

In the first interview, apathy and depressive moods were observed when she spoke about life events related to her friends at school and how this situation interfered with her functioning. Physical examination revealed a weight of 83.1 kg, height of 1.63 m, body mass index of 31.3, body mass index standard deviation score of 2.689, and blood pleasure of 120/80 mmHg. She denied muscular pain and no goiter or myxedema.

Soon after admission, a blood test and electrocardiogram (EKG) were performed. EKG, liver function tests, electrolytes, and whole blood count were within normal limits. Due to her isolation at home and selective food intake, levels of some vitamins were analyzed; vitamin D3 and folic acid levels were insufficient (vitamin D3: 20.9 ng/mL insufficient with levels above 30 ng/mL and folic acid: 2.3 ng/mL insufficient with levels above 3 ng/mL). The thyroid function test showed thyroid-stimulating hormone (TSH) levels of 24.159 mIU/L (reference range: 0.400–4.00), free thyroxine (FT4) 0.93 ng/dL (reference range: 0.80–2.00), and triiodothyronine (T3) 1.08 ng/mL (reference range: 0.70–1.90), with antithyroid peroxidase antibodies >1300 IU/mL (reference range: <35).

We requested interconsultation with the endocrinology department where hypothyroidism secondary to Hashimoto's thyroiditis was diagnosed, and treatment with levothyroxine 50 micrograms/day was recommended. We began folic acid (5 mg/day) and vitamin D (0.266 mg/alternate days) supplementation too.

During her hospital stay, the patient adapted well to the rules of the unit, was sociable with peers, and showed no behavioural disturbances at any time.

After 8 days in our acute unit, the patient showed significant improvement in mood and social interaction and was discharged. Monitoring continued in the outpatient clinic, while the patient maintained the same pharmacological treatment. At six weeks, the patient was asymptomatic; she had returned to school showing acceptable performance and socialization with family and classmates. The new thyroid function test showed TSH 9.096 mIU/L and FT4 1.21 ng/dL. Six-months after discharge, the improvement persisted, and thyroid function was TSH 6.935 mIU/L and FT4 1.35 ng/dL and antithyroid peroxidase antibodies decreased, with 75 micrograms/day of levothyroxine.

## 3. Discussion

This paper reports a case of hypothyroidism in a female adolescent who was attended for psychiatric symptoms. The literature highlights the importance of measuring thyroid function in patients with refractory mood disorders, rapid cycling, mixed episodes, and treatment with mood stabilizers (especially lithium) and in whom there is no improvement after treatment is initiated. In our department, thyroid function tests are routinely requested for adolescent patients with affective symptoms from the initial assessment whenever there are clinical manifestations of hypothyroidism such as decreased school performance, fatigue and sluggishness, emotional lability, and depressed or altered mood. This behavior may simulate other disorders of adolescence [4, 6]. The abnormalities found on physical examination include short stature, apparent excess weight (more fluid retention than obesity), having a puffy face with a dull, placid expression, bradycardia, pseudohypertrophy of the muscles, and delayed deep tendon reflexes. The thyroid gland may be normal in size, not palpable, or diffusely enlarged [7]. As for the laboratory analysis, determining the levels of TSH and FT4 is usually sufficient to make a diagnosis. Primary hypothyroidism presents high TSH and low free T4. Secondary hypothyroidism presents with low or normal TSH and decreased FT4. Autoimmune thyroiditis is confirmed by positive antithyroid antibodies (anti-TPO 85–90% higher) but its positivity does not imply hypothyroidism. [6]. In the National Health and Nutrition Examination Survey (NHANES III) from 1988 and 1994, 6.3 percent of adolescents (12 to 19 years of age) had positive antithyroglobulin antibodies (Tg Ab) and 4.8 percent had positive antithyroid peroxidase antibodies (TPO Ab) [8].

In paediatrics, the most common age of presentation of autoimmune thyroiditis is adolescence, though the disease may occur at any time even, in rare cases, in children under one year of age [9]. The initial presence of goiter and elevated thyroglobulin antibodies, the presence of celiac disease, and a progressive increase in thyroperoxidase antibodies and TSH value predict a progression toward overt hypothyroidism [10].

Thyroid hormones exert their influence on the central nervous system through a variety of mechanisms: modulation of gene expression of several groups of proteins, some of them with known physiopathological implications in mood disorders and the influence on serotonin and noradrenergic neurotransmission, known to be one of the modes of action of antidepressants [11].

T4 is the treatment of choice in children and adolescents with hypothyroidism. The goals of treatment are to restore normal growth and development, including pubertal development [7]. In patients who present with severe, longstanding hypothyroidism, slow correction with FT4 is advisable in order to minimize the potential development of unwanted side effects (deterioration in school performance, short attention span, hyperactivity, insomnia, and behavioral difficulties). In such patients, the replacement dose should be increased slowly over several weeks to months [12]. McClelland et al. recommended a period of closer observation before making any adjustments in the dose of thyroxine in order to detect noncompliance [13]. Several reports indicate that in hypothyroid patients the thyroid hormone replacement alleviates depressive symptoms, and thyroid supplements are accepted as an effective treatment option for affective disorders. The cumulative dose of levothyroxine over preceding weeks is best reflected in the serum TSH, and noncompliant patients require careful education as to the rationale for treatment [14]. In the patient we present, the TSH levels diminished after six weeks of treatment, which means there was adequate therapeutic compliance and hence clinical improvement.

The natural course of juvenile autoimmune thyroiditis is quite variable, and thyroid functions should be monitored periodically to detect hypothyroidism in all children and adolescents including those who are initially euthyroid or have subclinical hypothyroidism. Patients who are not receiving treatment at the time of presentation may require treatment subsequently. Levothyroxine therapy may have beneficial effects on the clinical course of the disease and on antibody titers [15].

Diagnosis of hypothyroidism is particularly important in adolescence because this condition retards growth in height and development of secondary sexual characteristics; delayed onset of puberty can also occur and diagnosing autoimmune thyroiditis (AT) at an early stage offers the opportunity for a timely intervention. Its potential association with papillary thyroid carcinoma is an additional reason for a careful follow-up of patients with AT [16].

## 4. Conclusions

Our case is an example of how neuropsychiatric symptoms in children and adolescents may have their etiology in an alteration of the functioning of the hypothalamic-pituitary-thyroid axis. After a proper diagnosis is made, treatment can focus on controlling the signs and symptoms presented, thus avoiding overmedication and preventing future complications in other body systems. Evaluation of thyroid functioning may be prudent in children and adolescents with neuropsychiatric symptoms, and we recommended including TSH and T4 blood levels in the complementary tests for differential diagnoses.

## Abbreviations

EKG: Electrocardiogram
TSH: Thyroid-stimulating hormone
FT4: Thyroxine
T3: Triiodothyronine.
